# Social perceptions and the stigmatization towards fifteen mental illnesses in France: a preliminary study on the role of vital force and burden

**DOI:** 10.3389/fpsyt.2024.1336690

**Published:** 2024-03-14

**Authors:** Michael Dambrun, Gaétan Marinho, Laurie Mondillon, Maélys Clinchamps, Frédéric Dutheil, Nadia Chakroun, Frédérique Teissedre, Ladislav Motak, Maya Corman, Isabelle Cuchet, Juliette Fargheot, Marie Izaute, Julia Daugherty, Roxane de la Sablonnière, Brittany L. Lindsay, Andrew C. H. Szeto

**Affiliations:** ^1^ Université Clermont Auvergne, LAPSCO CNRS, Clermont-Ferrand, France; ^2^ University of Calgary, Calgary, AB, Canada; ^3^ Université Clermont Auvergne, CNRS, LAPSCO, Physiological and Psychosocial Stress, CHU Clermont-Ferrand, WittyFit, Clermont-Ferrand, France; ^4^ Université de Montréal, Montréal, QC, Canada

**Keywords:** mental illnesses stigma, social rejection, vital force, burden, SUBAR, danger, communal/agentic traits

## Abstract

**Introduction:**

This study examined social perceptions and rejection towards fifteen mental illnesses, as well as a preliminary test of the SUBAR model, that hypothesized perceptions of both vital forces and burden would be negatively and positively related to social rejection, respectively.

**Methods:**

Using an online survey with participants from France (*n* = 952), social rejection was assessed using a feeling thermometer and a social distance scale, while social perceptions were measured using visual analog scales.

**Results:**

A stigma map for these different disorders is drawn up, revealing the social perceptions and levels of stigmatization specific to certain mental illnesses. Controlling for relevant social perceptions (i.e., danger, warmth, competence), we found that perception of burden was positively and significantly associated to social distance and negative feeling for 73% and 67% of mental illnesses, respectively. The perception of vital force was negatively and significantly related to social distance and negative feeling for 87% and 20% of mental illnesses, respectively. The change in *R2* between model 1 (i.e. perception of danger, warmth, competence) and model 2 (i.e. model 1 + perceptions of vital force and burden) significantly improved in 100% of cases for social distance and 67% of cases for negative feeling.

**Conclusion:**

These preliminary data provide support for the SUBAR model and call for further investigations to better understand the social rejection of people with mental illnesses.

## Introduction

People living with mental illnesses are subject to stigmatization, social rejection, and even dehumanization (1–6). However, not all mental illnesses are rejected to the same degree. For example, Marie et al. (Study 2) (3) revealed that the general population (in New Zealand) is significantly more likely to engage in an interpersonal relationship with a person labeled as having depression than with a person labeled as having schizophrenia. It appears that this difference in social distance is explained, at least in part, by an increased perception of dangerousness towards schizophrenia, which is consistent with theories indicating that threat leads to stigmatization and prejudice (7).

Duckitt’s dual-process cognitive-motivational model of ideology and prejudice proposed that two main pathways would lead to the stigmatization of certain social groups: on the one hand, perceptions of threat and danger and, on the other, dominance/subordination stakes (8). There is some support for this model in the context of schizophrenia (2); the more individuals perceive danger and favor social dominance, the more they stigmatize people with schizophrenia. A second theoretical model that appears relevant explaining the stigmatization of mental illnesses is the stereotype content model (9). This model is based on the premise that individuals are predisposed to (i) first assess a stranger’s intention to harm or help them (warmth dimension) and then (ii) judge the stranger’s ability to act on this perceived intention (competence dimension). The different combinations between these two dimensions reliably predict affective reactions towards a variety of social groups (10). Sadler et al. (11) found that the stereotype content (i.e. warmth and competence) underlying the stigmatization of mental illnesses is not the same for all disorders. For example, individuals with disorders associated with psychotic symptoms (e.g., schizophrenia) are perceived as hostile and incompetent, whereas those with disorders associated with neurocognitive deficits (e.g., Alzheimer) are only perceived as incompetent.

In addition to these models, the Social Utility-Based Acceptance/Rejection (SUBAR) Model (12) has recently been proposed to explain the emergence of stigmatization towards different social categories, including mental illnesses. This model offers a complementary explanation to previous models, which could help improve our understanding of stigmatization. In addition to the perception of dangerousness and the ability to carry out a negative intention (9), the SUBAR model proposes that stigmatization can also stem from the target’s perceived social utility. As this model has not yet been empirically tested, the current study was a preliminary test of the SUBAR model. This model proposes that human social cognition evaluates and reacts to agents/groups in a given social system on the basis of a social utility calculation aimed at determining whether individuals/groups contribute as much to the system as they benefit from it. To perform this calculation, individuals essentially dichotomize two perceived antagonistic forces: upward and downward forces. Upward forces are perceived *vital forces* (e.g., skills, resources, willpower), as they add value to a system and make a system more efficient in creating resources with positive social value. On the other hand, there are the downward forces, which are made up of perceived weaknesses that are likely to harm the system and weigh it down. This is the dimension of perceived *burden* (e.g., demotivation, use of benefits, dependence on others), which can fall on society or, in an interpersonal or family context, on the caregiver, for example. The model proposes that the result of the calculation predicts attitudes and behaviors towards the targets concerned. Overall, the perception of vital forces would promote the acceptance of the target agents/groups (i.e., positive attitudes and behaviors), as those ranked high in this dimension are perceived as contributing positively to the given social system. Conversely, the perception of a burden on others and/or society would promote the rejection of the target (i.e. negative attitudes and behaviors towards it), and therefore its stigmatization.

This new model leads to the prediction that mental illnesses would be associated with varying degrees of vital force and burden, which may explain why some disorders are more stigmatized than others. Firstly, we predicted that certain disorders, such as alcohol addiction and schizophrenia (highly stigmatized mental disorders (13);), would be associated with low vital force and high perceived burden. This would be less the case for other disorders that are stigmatized to a lower degree, such as eating disorders, obsessive compulsive-disorder (OCD) or anxiety, for example (11). Secondly, we predicted that the perceptions of vital force and burden will predict stigmatization. Specifically, we hypothesized that perceptions of vital force will be negatively and significantly related to stigmatization. Conversely, the perception of burden should correlate positively and significantly with social rejection. To test our predictions, we assessed negative feeling (i.e. emotional response) and social distance (i.e. a proxy measure of behavioral rejection/discrimination) towards 15 different mental illnesses. To test the added statistical contribution of the SUBAR model to the explanation of stigma, we compared model 1 (i.e. perceptions of danger, warmth, and competence) to model 2 (i.e. model 1 + perceptions of vital force and burden) by computing the change in *R* ^2^ in a two-step multiple linear regression procedure.

## Method

### Participants

One thousand and sixty French citizens opened the online questionnaire, with 952 completing at least 60% of the questionnaire. The inclusion criterion was simply having answered all the questions for a single disorder. Thus, the statistical analyses included 952 participants. Of these 952 participants, 487 completed the entire questionnaire, including the demographic questions at the end. Among these 487 participants, 70.2% were women (*N* = 342), 25.9% were men (*N* = 126), and 3.9% were another gender (*N* = 19). The average age of the sample was 21.0 years (*SD* = 6.0; minimum = 18; maximum = 59). Most of the participants were University students from various fields (92%), with 10% of the sample being psychology students (*N* = 50). The study was approved by local Ethics Committee (IRB00013412, “CHU de Clermont Ferrand IRB #1”, IRB number 2022-CF061) with compliance to the French policy of individual data protection. All participants have given informed consent to participate in the research.

## Materials

### Social distance

We used the social distance scale from Mather, Jones, and Moats (14) as a proxy measure of behavioral rejection/discrimination (15). Of the eight original items, we selected the four items that were most relevant for the context of mental illnesses, as well as to increase brevity. The four selected items were: (1) “I would be willing to accept a person with [a X disorder] as a close relative by marriage”; (2) “I would be willing to accept a person with [a X disorder] as a close friend”; (3) “I would be willing to accept a person with [a X disorder] as a neighbor on the same street”; and (4) “I would be willing to accept a person with [a X disorder] as a coworker”. For each item, participants indicated their level of agreement with each statement using a Visual Analog Scale (VAS) ranging from (0) strongly disagree to 100 (strongly agree). We followed the recommendations of Mather et al. (14) and computed an intensity score (iScore) by multiplying Item 1 by 1, Item 2 by 2, Item 3 by 3 and Item 4 by 4. We divided this score by 10 to obtain a mean score with a minimum of 0 and a maximum of 100. This score was then subtracted from 100, so that a higher score indicates greater social distance (global Cronbach *α* = 0.89; global McDonald *ω* = 0.89).

### Feeling thermometer

Negative feeling (i.e. prejudice) was assessed using a VAS ranging from 0 (very negative) to 100 (very positive). Participants had to indicate their general attitude towards adults’ people with a X disorder. The score on this scale was reversed coded. Thus, a higher score indicated a greater negative feeling.

### Social perceptions: vital forces/burden, warmth/competence and dangerousness

For each item, participants had to answer with a VAS ranging from 0 (strongly disagree) to 100 (strongly agree) the extent to which they personally perceived that adults with a X disorder are: (1) “able to occupy a position of high status and responsibility in society” (perception of vital force); (2) “a drag on society” (perception of burden); (3) “dangerous” (perception of dangerousness); (4) “friendly, sociable, warm” (perception of sociability); (5) “moral, honest, sincere” (perception of morality); (6) “competent, intelligent, efficient” (perception of ability); (7) “ambitious, self-confident, persevering” (assertive dimension). The last four items grouped together three traits each, to keep the questionnaire as short as possible. A score of “warmth/communal-traits” was computed by averaging sociability and morality (global Cronbach *α* = 0.85; global McDonald *ω* = 0.85). A score of “competence/agentic-traits” was computed by averaging the ability and assertive dimensions (global Cronbach *α* = 0.79; global McDonald *ω* = 0.79). For a similar methodology, see Aubé, Rohmer, and Yzerbyt (16).

### Procedure

The Qualtrics online platform was used to deploy this online questionnaire. Participants were contacted by email via the university’s mailing list to participate in the study. Once providing consent, participants completed the social perceptions, negative feelings, and social distance questions towards five randomly assigned mental illnesses out of a total of 15 (i.e., Attention deficit hyperactivity disorder – ADHD, Alcohol addiction, Anorexia, Autism spectrum disorder – ASD, Bipolar disorder, Bulimia, Burnout, Depressive disorder, Digital addiction, Gender dysphoria, Generalized anxiety disorder - GAD, Obsessive-compulsive disorder – OCD, Post-traumatic stress disorder – PTSD, Schizophrenia, Suicidal thoughts and behaviors) that appear in the Diagnostic and Statistical Manual of Mental Disorders (5^th^ Ed, DSM-5; American Psychiatric Association, 2013). Participants only completed the assessments for five of the mental illnesses, as opposed to 15, to keep the questionnaire brief. The order of the measures (i.e., social perceptions, negative feeling, and social distance) and the order of items for each measure were also randomized. The number of participants that responded to each mental disorder is presented as **Supplementary Material in Table S1**. Based on *a priori* power analysis using G*Power 3.1 (alpha = 0.05, Power = 95%, expected *r* = 0.30), we had planned for a minimum of 138 participants per mental disorder. This was achieved, with a minimum of 176 and a maximum of 203 participants per disorder.

### Statistical analysis

First, for the descriptives, we calculated the means for each measure and for each mental disorder. In order to compare mental illnesses with one another, we also calculated the grand mean for all illnesses. To enhance comprehension of the main results, these descriptives are presented graphically. As participants were randomly assigned to only 5 mental illnesses out of a total of 15, it was not possible to conduct cluster analyses. Thus, groupings were based on the grand means. Next, we tested our main hypothesis concerning the relationships between different social perceptions, negative feeling, and social distance. We thus conducted a series of correlational analyses. As most social perceptions did not follow a normal distribution, we performed Spearman correlations. Next, we performed a series of multiple regression analyses to identify the robust relationships between social perceptions and both negative feeling and social distance. Using a two-step multiple linear regression procedure, we computed the change in *R* ^2^ between model 1 (perceptions of danger, warmth and competence) and model 2 (i.e. model 1 + perceptions of vital force and burden). There was no multicollinearity (i.e., all *VIF*s < 3). We calculated the cook distance for each analysis and found that no extreme values were present. The normality test rejected the normality hypothesis most of the time (i.e., failed to achieve statistical normality). For this reason, we conducted bootstraps (i.e. bootstrapping based on 5000 replicates).

## Results

### Descriptives

#### Social distance and negative feeling

**Figure 1:**
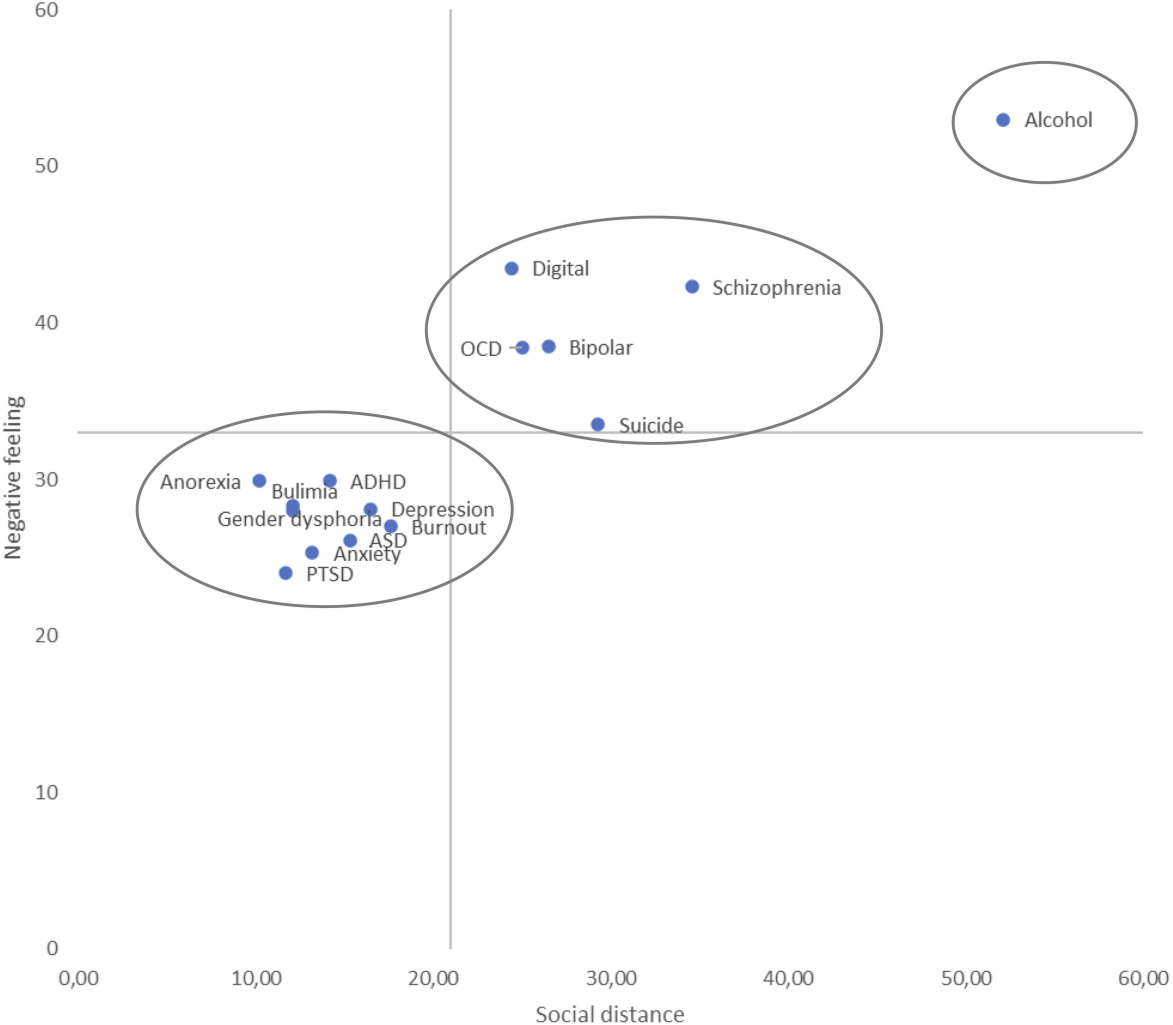
Social distance and negative feeling for fifteen mental illnesses in France.

The grand mean of social distance for the 15 mental illnesses was 21.0 (see X-axis on **Figure 1**) and the grand mean of negative feeling was 33.05 (see Y-axis on **Figure 1**). The mean scores of social distance and negative feeling for each mental disorder are displayed on **Figure 1**. Alcohol addiction was the most rejected mental disorder follow by a group of five mental illnesses (i.e., schizophrenia, digital addiction, bipolar disorder, OCD, and suicidal thoughts and behaviors). The other mental illnesses were rated more favorably.

#### Vital force and burden

**Figure 2:**
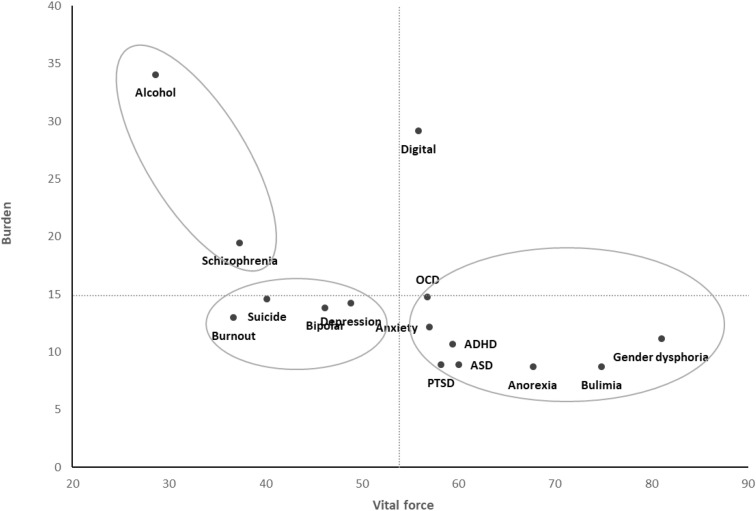
Perceptions of vital force and burden for fifteen mental illnesses in France.

The grand mean of vital force for the fifteen mental illnesses was 53.9 (see X-axis on **Figure 2**) and the grand mean of burden was 14.8 (see Y-axis on **Figure 2**). The vital force/burden means for each mental disorder are presented in **Figure 2**. There were three groups: the low vital force/high burden group (i.e., alcohol addiction and schizophrenia), the high vital force/low burden group (i.e., gender dysphoria, bulimia, anorexia, ASD, ADHD, PTSD, generalized anxiety disorder and OCD). An intermediate group characterized by low vital force/intermediate burden was constituted of four mental illnesses (i.e., burnout, suicidal thoughts and behaviors, bipolar disorder, and depressive disorder). Digital addiction did not align with other conditions, with a high level of burden and an intermediate level of vital force.

#### Warmth and competence

**Figure 3:**
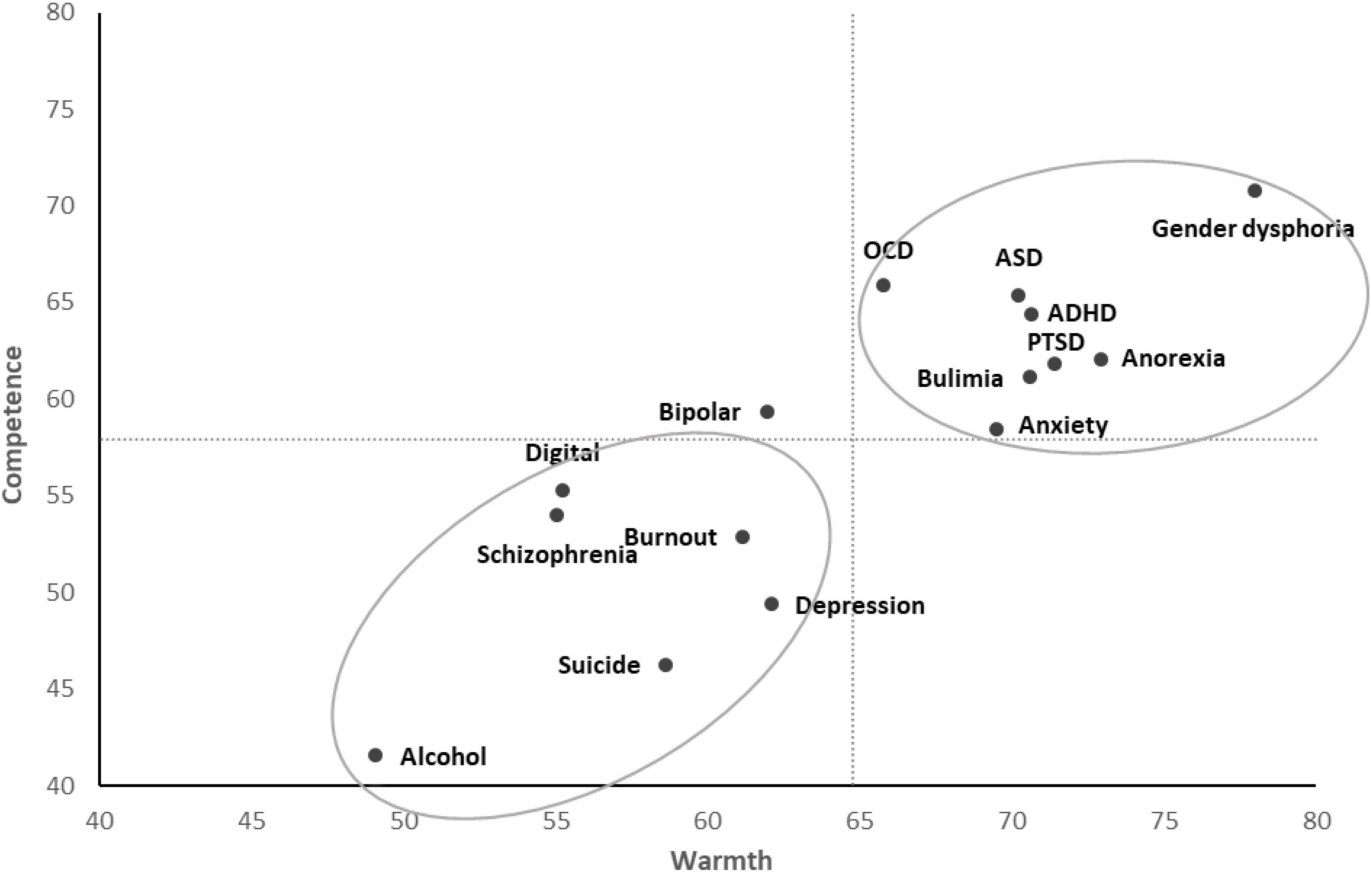
Perceptions of warmth/communal and competence/agentic for fifteen mental illnesses in France.

The grand mean of warmth for the fifteen mental illnesses was 64.8 (see X-axis on **Figure 3**) and the grand mean of competence was 57.9 (see Y-axis on **Figure 3**). The warmth/competence means for each mental disorder are shown in **Figure 3**. There are two main groups. First, there is a low warmth/low competence group in which there are six mental illnesses (i.e. alcohol addiction, suicide thoughts and behaviors, schizophrenia, digital addiction, depression and burnout). The second group included the nine other mental illnesses (i.e. generalized anxiety - GAD, bulimia, PTSD, anorexia, ADHD, OCD, ASD, and gender dysphoria) and corresponds to the high warmth/high competence combination. The most stigmatized groups on the warmth and competence dimensions were alcohol addiction and suicidal thoughts and behaviors. Gender dysphoria was the one rated most favorably on these dimensions.

#### Dangerousness

The grand mean of dangerousness for the fifteen mental illnesses was 22.7, with five groups above this score: alcohol addiction (62.7), schizophrenia (41.2), bipolar disorder (35.5), suicide thoughts and behaviors (33.4) and OCD (26.4). All the means are presented in **Supplementary Materials** (see **Table S2**).

#### Relationships between social perceptions and rejection

Zero-order Spearman correlations between social perceptions and the rejection measures (i.e., social distance and negative feeling) are presented in **Supplementary Materials** (see **Tables S3**, **S4**). While vital force (*Rho* ranged from -0.18 to -0.60), warmth/communal (*Rho* ranged from -0.29 to -0.57) and competence/agentic (*Rho* ranged from -0.29 to -0.53) were negatively and significantly related to both social distance and negative feelings, burden (*Rho* ranged from 0.20 to 0.48) and dangerousness (*Rho* ranged from 0.10 to 0.52) were positively related to these outcomes positively.

We conducted a series of multiple regression analyses. The bootstrapped unstandardized beta coefficients of the relationships between the various social perceptions, social distance and negative feeling are presented in **Table 1**. To summarize the results, we computed to percentage of significant relations for each social perception across the fifteen mental illnesses. Concerning social distance, the most frequently related social perceptions were vital force (87%), burden (73%), danger (60%) and warmth traits (53%). Competence traits were not frequently related to social distance (7%). Adding vital force and burden (model 2) significantly improved the percentage of explained variance in 100% of cases (see *R* ^2^ change in **Table 1**). The average *R* ^2^ change was 0.075 (average total adjusted *R* ^2 =^ 0.37). Concerning negative feeling, the most frequently related social perceptions were warmth traits (80%) and burden (67%). Danger (27%), vital force (20%) and competence traits (7%) were less frequently related to negative feeling. Change in *R* ^2^ between model 1 and 2 was significant for 10 out of the 15 mental illnesses (67%). The average *R* ^2^ change was 0.037 (average total adjusted *R* ^2 =^ 0.26).

**Table 1:** Multiple linear regression bootstrapped unstandardized beta coefficients of the relationships between various social perceptions and social distance (A), and negative feeling (B) and R^2^ change between model 1 and model 2**^+^**.

	*Multiple independent variables*								
	Vital force		Burden	Danger	Warmth/ Communal	Competence/ Agentic	R^2^ change model1/model2^+^	Total adjusted R^2^	
A. Social distance towards									
1. Attention deficit hyperactivity disorder-ADHD	-0.11**		0.32***	0.16*	-0.15*	-0.06	0.10***	0.43
2. Alcohol addiction	-0.28***		0.15*	0.24**	-0.14	-0.18	0.06***	0.38
3. Anorexia	-0.08**		0.18*	-0.06	-0.10*	-0.02	0.09***	0.21
4. Autism spectrum disorder - ASD	-0.26***		0.15	0.24***	-0.05	-0.05	0.14***	0.43
5. Bipolar disorder	-0.29***		0.13	0.16**	-0.22*	-0.10	0.09***	0.43
6. Bulimia	-0.10*		0.13*	0.20**	-0.28***	0.06	0.04**	0.36
7. Burnout	-0.05		0.14	-0.04	-0.10	-0.16*	0.03*	0.18
8. Depressive disorder	-0.10*		0.20*	0.20***	-0.15*	0.00	0.06***	0.43
9. Digital addiction	-0.25***		0.15**	0.08	-0.24**	0.00	0.09***	0.40
10. Gender dysphoria	-0.15***		0.16*	0.16	-0.09	-0.05	0.09***	0.51
11. Generalized anxiety disorder	-0.14**		0.15*	0.10	-0.08	-0.07	0.07***	0.22
12. Obsessive-compulsive disorder - OCD	-0.10		0.12	0.27***	-0.33**	-0.03	0.02*	0.46
13. Post-traumatic stress disorder - PTSD	-0.12**		0.26***	0.18***	0.01	-0.09	0.07***	0.33
14. Schizophrenia	-0.22**		0.24***	0.27***	-0.19*	-0.14	0.08***	0.50
15. Suicidal thoughts and behaviors	-0.17*		0.24*	0.07	-0.08	-0.19	0.10***	0.26
***% of significant relations***	***87%***		***73%***	***60%***	***53%***	***7%***		
B. Negative feeling towards									
1. Attention deficit hyperactivity disorder-ADHD	0.08		0.25***	0.05	-0.40***	0.09	0.05**	0.24
2. Alcohol addiction	0.01		0.13**	0.14**	-0.20*	-0.15	0.03*	0.32
3. Anorexia	0.03		0.05	0.10	-0.29**	-0.08	0.00	0.19
4. Autism spectrum disorder - ASD	-0.02		0.25**	0.05	-0.25**	-0.19*	0.03*	0.27
5. Bipolar disorder	0.05		0.13	0.07	-0.30***	-0.10	0.02	0.25
6. Bulimia	-0.15*		0.18**	0.10	-0.24*	-0.03	0.05***	0.30
7. Burnout	-0.04		0.19**	-0.0	-0.19*	-0.08	0.04**	0.19
8. Depressive disorder	-0.07		0.18*	0.04	-0.18*	-0.08	0.04*	0.22
9. Digital addiction	-0.19***		0.12**	0.06	-0.20*	-0.07	0.07***	0.36
10. Gender dysphoria	-0.22**		0.17*	0.14	-0.23*	0.02	0.07***	0.36
11. Generalized anxiety disorder	-0.09		0.07	0.21*	-0.24**	0.00	0.02	0.19
12. Obsessive-compulsive disorder – OCD	-0.08		0.04	0.07	-0.34***	-0.04	0.01	0.37
13. Post-traumatic stress disorder – PTSD	-0.02		0.12	0.15*	-0.22	-0.15	0.01	0.23
14. Schizophrenia	-0.05		0.22***	0.15**	-0.11	0.05	0.08***	0.27
15. Suicidal thoughts and behaviors	0.04		0.19*	0.00	-0.20	-0.17	0.03*	0.17
***% of significant relations***	***20%***		***67%***	***27%***	***80%***	***7%***		

*** p < 0.001; ** p < 0.01; * p < 0.05; **^+^** R^2^ change between model 1 (danger, warmth, competence) and model 2 (danger, warmth, competence, vital force and burden). The bold values corresponds to the % of significant relations among the 15 disorders for each variables.

## Conclusion

This study revealed some important results concerning stigmatization towards different mental illnesses in France and the SUBAR model. Firstly, the results reveal that of the 15 mental illnesses investigated, alcohol addiction was by far the most stigmatized in terms of social distance and negative feeling. When we examined communal/agentic and vital force/burden perceptions, once again the disorder that was perceived least favorably was alcohol dependence. This is consistent with the literature review by Schomerus et al. (13), who concluded, based on several surveys carried out in different parts of the world, that alcohol addiction “is a particularly severely stigmatized mental disorder” (p. 105).

On the dimensions of warmth and competence, Sadler et al. (11) found that the mental disorders rated most favorably were eating disorders, OCD, anxiety, bipolar disorder, and depression. Some similarities were found for the first three, but depression and bipolar disorder seem to be rated less favorably on these dimensions in France. Concerning the most stigmatized, in addition to alcohol addiction, we also found schizophrenia and other mental illnesses that were not investigated in Sadler’s study, such as suicide, burnout, and digital addiction. So, there seems to be some cultural variation.

This study provides an initial mapping of perceptions of mental illnesses in relation to the SUBAR model dimensions of vital force and burden. As expected, the two most stigmatized mental disorders (alcohol addiction and schizophrenia) were in the area associating a low level of vital force and a high level of burden. In other words, they are depreciated on both dimensions. Conversely, low-stigma groups such as eating disorders, gender dysphoria, PTSD, and autism were positively evaluated on both dimensions (i.e., low burden/high life force). Lastly, some groups were depreciated only on the dimension of vital force, but not on the dimension of burden. These were moderately stigmatized mental illnesses, such as burnout, suicide, depression, and bipolar disorder. Those in society may not perceive those with these specific mental illnesses as having the ability to obtain high status or responsibilities, but are not necessarily perceiving them as a burden to society (e.g., on the healthcare system). Only one disorder, digital addiction, was depreciated solely on the burden dimension (but not on the life-force dimension), making this a unique situation requiring further investigation to understand this outcome. The study population consisted overwhelmingly of students who are regularly exposed to excessive screen use (17). Perhaps familiarity with this disorder would partly explain its stigmatization on the dimension of burden alone. In a broad perspective, this would be consistent with the perspective developed by Corrigan and Nieweglowski (18), who proposed that familiarity with a disorder can sometimes increase its stigmatization, particularly when this is underpinned by a perception of burden for caregivers. Here, we are not talking about caregivers, but about people exposed to the interpersonal constraints exerted by excessive screen use. This seems to suggest that the burden dimension can be relevant to different contexts (13).

The analysis of the relationships between social perceptions and the stigmatization of mental illnesses provides preliminary support for the SUBAR model (12). Indeed, not only do bivariate correlations reveal significant relations of moderate size in most cases, but more importantly, when statistically controlling for perceptions of dangerousness and warmth/competence traits (factors known to predict the stigmatization of mental illnesses), perceptions of burden and vital force remained significantly predictive of stigmatization for a significant number of mental illnesses. As expected, while perception of burden was positively related to stigmatization, assessed by social distance and negative feeling, perception of vital force was most negatively related to social distance. The results also confirm that perceptions of danger and warmth/communal (but not competence/agentic) are robust predictors of stigmatization, but sometimes less so than perceptions of vital force and burden, particularly in the case of social distance. While social distance is considered a behavioral proxy for rejection/discrimination, negative feeling is a measure of prejudice (emotional response). Research suggests that emotional response to mental illness predates rejection/discrimination (15). In the present research, while the perception of warmth seems closely related to the “like-dislike” emotional response, the intention to reject and discriminate (a variable with potentially important social consequences) is more closely related to perceptions derived from the SUBAR model. Further investigation is required for these findings, specifically exploring if the SUBAR model yields distinct predictions for stereotypes, prejudice, and behavioral intentions (variables which typically exhibit weak correlations) (19).

In addition, comparisons between model 1 (i.e., perception of danger, “communal-traits”, “agentic-traits”) and model 2 (i.e. model 1 + perceptions of vital force and burden) revealed significant improvement in 100% of cases for social distance and 67% of cases for negative feeling. This suggests that SUBAR model could make an additional contribution to explaining the stigmatization of mental illnesses. Apart from the sense of threat (7) elicited by a target (perception of danger) and the perception of a target’s capability to enact a negative intention toward us (stereotype content model (9)), stigmatization—particularly in the context of mental illness—may also stem from a perception of low social utility. A wealth of research reveals that two main dimensions are involved in the perception of other people and social groups: *agentic content*, which refers to goal achievement and task functioning (competence, assertiveness, decisiveness), and *communal content*, which has a social function of maintaining relationships and facilitating positive social interactions (e.g. helpfulness, benevolence, trustworthiness). These two dimensions have been described as “fundamental” or the “Big Two” (20–24). Although there are links, the SUBAR model also posits that individuals within a specific social system engage in a calculation to assess the contributions of others to the system. This model proposes that this utility calculus is the result of two dimensions: the perception of vital force and the perception of burden, which do not seem to be reducible to the agentic and communal dimensions. According to a recent literature review (12), the emphasis on perceived social utility is primarily linked to perceptions of a target’s efficacy, dynamism, and confidence. On the other hand, perceived burden is primarily attributed to perceptions of fragility/vulnerability, a tendency to demotivate, and a propensity to depend on others. Of course, future research may test these hypotheses.

This preliminary study has several limitations. The first limitation concerns the study sample, which consisted mainly of female French students. Replication with a more heterogeneous and culturally diverse sample would be welcome, especially considering age and gender can play a role in mental illness stigma (25). Secondly, although it has been shown that a single item can have similar psychometric qualities to a scale made up of several items (26–28), we think that it would be important to develop, in a future study, a scale assessing vital force and burden made up of items assessing different aspects of these perceptions. It is unlikely, for example, that the item used in the present study to assess the perception of vital force would cover all aspects of this construct. Thirdly, warmth and competence (mean *r* = 0.73) on one hand, and vital force and burden on the other hand (mean *r* = -0.34), are not independent constructs. They shared a common variance (see **Table S5 in Supplementary Materials**). Thus, caution should be exercised when interpreting our figures with two right-angles. In sum, while these results are encouraging for the SUBAR model, further research is needed.

## Data availability statement

The original contributions presented in the study are included in the article/**Supplementary Materials**, further inquiries can be directed to the corresponding author/s.

## Ethics statement

The studies involving humans were approved by IRB00013412, “CHU de Clermont Ferrand IRB #1”, IRB number 2022-CF061. The studies were conducted in accordance with the local legislation and institutional requirements. The participants provided their written informed consent to participate in this study.

## Author contributions

MD: Conceptualization, Data curation, Formal Analysis, Investigation, Methodology, Project administration, Software, Supervision, Validation, Writing – original draft, Writing – review & editing. GM: Data curation, Formal Analysis, Investigation, Methodology, Software, Writing – original draft, Writing – review & editing, Validation. LMn: Conceptualization, Investigation, Methodology, Writing – review & editing, Resources, Validation. MCl: Investigation, Methodology, Project administration, Writing – review & editing. FD: Conceptualization, Investigation, Methodology, Project administration, Writing – review & editing. NC: Conceptualization, Methodology, Writing – review & editing. FT: Conceptualization, Methodology, Writing – original draft, Writing – review & editing. LMt: Conceptualization, Methodology, Writing – original draft, Writing – review & editing. MCo: Conceptualization, Methodology, Resources, Writing – review & editing. IC: Conceptualization, Methodology, Resources, Writing – review & editing. JF: Formal Analysis, Investigation, Software, Writing – review & editing. MI: Resources, Writing – review & editing. JD: Resources, Writing – original draft, Writing – review & editing. RdlS: Resources, Writing – original draft, Writing – review & editing. BL: Resources, Writing – original draft, Writing – review & editing. AS: Resources, Writing – original draft, Writing – review & editing.
