# Supplementary material for: Social perceptions and the stigmatization towards fifteen mental illnesses in France: a preliminary study on the role of vital force and burden
Source: Front Psychiatry. 2024 Mar 14;15:1336690. doi: 10.3389/fpsyt.2024.1336690 (PMC10973838; doi:10.3389/fpsyt.2024.1336690)
Supplement: Supplementary file 1 [file Table_1.docx]

SUPPLEMENTAL MATERIAL

Table S1. Number of participants by mental illness

|  | *N* |
| --- | --- |
| 1. Attention deficit hyperactivity disorder-ADHD | 203 |
| 2. Alcohol addiction | 177 |
| 3. Anorexia | 198 |
| 4. Autism spectrum disorder – ASD | 189 |
| 5. Bipolar disorder | 176 |
| 6. Bulimia | 185 |
| 7. Burnout | 193 |
| 8. Depressive disorder | 203 |
| 9. Digital addiction | 201 |
| 10. Gender dysphoria | 193 |
| 11. Generalized anxiety disorder - GAD | 188 |
| 12. Obsessive-compulsive disorder - OCD | 181 |
| 13. Post-traumatic stress disorder – PTSD | 194 |
| 14. Schizophrenia | 189 |
| 15. Suicidal thoughts and behaviors | 195 |

Table S2. Means of dangerousness by mental illness

|  | Perception of dangerousness |
| --- | --- |
| 1. Attention deficit hyperactivity disorder-ADHD | 14.8 |
| 2. Alcohol addiction | 62.7 |
| 3. Anorexia | 10.7 |
| 4. Autism spectrum disorder - ASD | 12.2 |
| 5. Bipolar disorder | 35.5 |
| 6. Bulimia | 8.4 |
| 7. Burnout | 14.5 |
| 8. Depressive disorder | 20.2 |
| 9. Digital addiction | 21.9 |
| 10. Gender dysphoria | 7.5 |
| 11. Generalized anxiety disorder - GAD | 11.9 |
| 12. Obsessive-compulsive disorder - OCD | 26.4 |
| 13. Post-traumatic stress disorder – PTSD | 19.5 |
| 14. Schizophrenia | 41.2 |
| 15. Suicidal thoughts and behaviors  *Grand Mean* | 33.4  22.7 |

Table S3. Zero-order Spearman correlations between various social perceptions and social distance towards fifteen mental illnesses.

|  | *Social perceptions* | | | | |
| --- | --- | --- | --- | --- | --- |
|  | Vital force | Burden | Danger | Warmth/  Communal | Competence/  Agentic |
| *Social distance* |  |  |  |  |  |
| 1. Attention deficit hyperactivity disorder-ADHD | -0.53*** | 0.42*** | 0.48*** | -0.42*** | -0.42*** |
| 2. Alcohol addiction | -0.46*** | 0.43*** | 0.43*** | -0.46*** | -0.48*** |
| 3. Anorexia | -0.35*** | 0.24*** | 0.10 | -0.40*** | -0.37*** |
| 4. Autism spectrum disorder - ASD | -0.57*** | 0.44*** | 0.41*** | -0.31*** | -0.38*** |
| 5. Bipolar disorder | -0.53*** | 0.42*** | 0.46*** | -0.46*** | -0.38*** |
| 6. Bulimia | -0.44*** | 0.39*** | 0.30*** | -0.48*** | -0.42*** |
| 7. Burnout | -0.31*** | 0.34*** | 0.17* | -0.36*** | -0.39*** |
| 8. Depressive disorder | -0.46*** | 0.46*** | 0.48*** | -0.43*** | -0.41*** |
| 9. Digital addiction | -0.58*** | 0.48*** | 0.40*** | -0.53*** | -0.52*** |
| 10. Gender dysphoria | -0.60*** | 0.47*** | 0.34*** | -0.40*** | -0.49*** |
| 11. Generalized anxiety disorder - GAD | -0.38*** | 0.28*** | 0.23*** | -0.29*** | -0.37*** |
| 12. Obsessive-compulsive disorder - OCD | -0.55*** | 0.45*** | 0.54*** | -0.57*** | -0.43*** |
| 13. Post-traumatic stress disorder – PTSD | -0.36*** | 0.41*** | 0.37*** | -0.30*** | -0.31*** |
| 14. Schizophrenia | -0.52*** | 0.47*** | 0.52*** | -0.54*** | -0.53*** |
| 15. Suicidal thoughts and behaviors | -0.46*** | 0.39*** | 0.34*** | -0.43*** | -0.45*** |

*Note*: *** *p* < 0.001; ** *p* < 0.01; * *p* < 0.05

Table S4. Zero-order Spearman correlations between various social perceptions and negative feeling towards fifteen mental illnesses.

|  | *Social perceptions* | | | | |
| --- | --- | --- | --- | --- | --- |
|  | Vital force | Burden | Danger | Warmth/  Communal | Competence/  Agentic |
| *Negative feeling thermometer* |  |  |  |  |  |
| 1. Attention deficit hyperactivity disorder-ADHD | -0.18* | 0.36*** | 0.26*** | -0.46*** | -0.30*** |
| 2. Alcohol addiction | -0.25*** | 0.40*** | 0.35*** | -0.42*** | -0.38*** |
| 3. Anorexia | -0.21** | 0.20** | 0.19** | -0.44*** | -0.36*** |
| 4. Autism spectrum disorder - ASD | -0.28*** | 0.35*** | 0.29*** | -0.45*** | -0.41*** |
| 5. Bipolar disorder | -0.19** | 0.31*** | 0.28*** | -0.44*** | -0.29*** |
| 6. Bulimia | -0.43*** | 0.31*** | 0.24** | -0.45*** | -0.41*** |
| 7. Burnout | -0.23*** | 0.34*** | 0.18* | -0.40*** | -0.35*** |
| 8. Depressive disorder | -0.37*** | 0.35*** | 0.28*** | -0.41*** | -0.38*** |
| 9. Digital addiction | -0.47*** | 0.36*** | 0.31*** | -0.40*** | -0.40*** |
| 10. Gender dysphoria | -0.50*** | 0.40*** | 0.30*** | -0.43*** | -0.43*** |
| 11. Generalized anxiety disorder - GAD | -0.30*** | 0.27*** | 0.34*** | -0.34*** | -0.30*** |
| 12. Obsessive-compulsive disorder - OCD | -0.43** | 0.31*** | 0.36*** | -0.54*** | -0.43*** |
| 13. Post-traumatic stress disorder – PTSD | -0.31*** | 0.36*** | 0.35*** | -0.42*** | -0.39*** |
| 14. Schizophrenia | -0.31*** | 0.45*** | 0.40*** | -0.33*** | -0.30*** |
| 15. Suicidal thoughts and behaviors | -0.21** | 0.33*** | 0.21** | -0.36*** | -0.34*** |

*Note*: *** *p* < 0.001; ** *p* < 0.01; * *p* < 0.05

Table S5. Correlations between communal/warmth and agentic/competence, and between vital force and burden

|  | **Correlations between** | |
| --- | --- | --- |
|  | **communal/warmth and agentic/competence** | **vital force and burden** |
| 1. Attention deficit hyperactivity disorder-ADHD | 0.77*** | -0.40*** |
| 2. Alcohol addiction | 0.72*** | -0.32*** |
| 3. Anorexia | 0.70*** | -0.30*** |
| 4. Autism spectrum disorder - ASD | 0.66*** | -0.25*** |
| 5. Bipolar disorder | 0.69*** | -0.38*** |
| 6. Bulimia | 0.79*** | -0.39*** |
| 7. Burnout | 0.72*** | -0.29*** |
| 8. Depressive disorder | 0.75*** | -0.38*** |
| 9. Digital addiction | 0.75*** | -0.35*** |
| 10. Gender dysphoria | 0.80*** | -0.63*** |
| 11. Generalized anxiety disorder | 0.69*** | -0.20** |
| 12. Obsessive-compulsive disorder - OCD | 0.71*** | -0.44*** |
| 13. Post-traumatic stress disorder - PTSD | 0.69*** | -0.20** |
| 14. Schizophrenia | 0.71*** | -0.29*** |
| 15. Suicidal thoughts and behaviors | 0.74*** | -0.29*** |
| ***Mean correlation across the 15 mental illnesses*** | *0.73* | *-0.34* |

*Note*: *** *p* < 0.001; ** *p* < 0.01; * *p* < 0.05
